# Supplementary material for: Conceptualizing safer sex in a new era: Risk perception and decision-making process among highly sexually active men who have sex with men
Source: PLOS Glob Public Health. 2022 May 6;2(5):e0000159. doi: 10.1371/journal.pgph.0000159 (PMC10022342; doi:10.1371/journal.pgph.0000159)
Supplement: S1 File — (DOCX) [file pgph.0000159.s001.docx]

SUPPLEMENTARY: INTERVIEW GUIDE IN SWEDISH AND WITH ENGLISH TRANSLATION

Hur gammal är du?

Bor du i Berlin eller är du här regelbundet?

Lever du någon form av relation?

Vad har du för utbildning?

Vad har du för sysselsättning?

Lever du med hiv?

*How old are you?*

*Do you live in Berlin or are you here on a regular basis.*

*Are you in a relationship?*

*What is your highest education?*

*What is your current occupation?
Do you live with HIV?*

SEX

För vilka syften träffar du killar eller män just nu?

Hur mycket sex har du?

*For what purposes do you meet men right now*

*How many sexual partners do you have?*

HIV AND SAFER SEX

Hur tänker du kring hiv och könssjukdomar?

Vad har du för tankar och upplevelse av kondomanvändande?

Praktiserar du säkrare sex?

Har ditt kondomanvändande förändrats över tid?

Finns det sammanhang då du har sex utan kondom?

Vad har du för inställning till riskreducerande strategier som oskyddat samlag med ejakulation utanför kroppen?

Känner du till vad PrEP är?

Har du kommit i kontakt med PrEP?

Hur tänker du kring att själv använda PrEP?

Hur tänker du kring andras användande av PrEP?

*What are your though about HIV and sexual transmitted infections?
What are your thoughts and experience of condom use?
Do you practice any kind of safer sex?*

*Have your condom practice changed over time?*

*Are there any specific situations where you do not have sex without a condom?*

*What are you views on to risk reduction strategies, for instance condomless sex with ejaculate outside the body?*

*Are you aware of the concept of PrEP?*

*Have you been in situation where PrEP has been discussed?*

*How do you feel about using PrEP?*

*How do you perceive other’s use of PrEP?*

TESTING

Har du testat dig för hiv eller en könssjukdom?

Har du testat positivt för någon könssjukdom?

Vad var anledningen till ditt senaste test?

*Have you ever tested for HIV or a sexually transmitted infection?
Have you ever tested positive for a sexually transmitted infection?*

*What was the reason behind your last test?*

LIVING WITH HIV

Är hiv något som diskuteras i samband med en dejt?

Hur tänker du på hiv i relation till smittsamhet?

*Is HIV something that is being discussed when dating?*

*What are your perception of undetectable = untransmittable?*
