# Supplementary material for: Conceptualizing safer sex in a new era: Risk perception and decision-making process among highly sexually active men who have sex with men
Source: PLOS Glob Public Health. 2022 May 6;2(5):e0000159. doi: 10.1371/journal.pgph.0000159 (PMC10022342; doi:10.1371/journal.pgph.0000159)
Supplement: Attachment — Submitted filename: Respons_letter.docx [file pgph.0000159.s002.docx]

**EDITOR’S NOTES**

Thank you so much for giving us the opportunity to improve the manuscript…

1. Please include a copy of the interview guide used in the study, in both the original language and English, as Supporting Information, or include a citation if it has been published previously.

*Response: Thanks, the interview guide has been translated from Swedish to English and is now included in the re-submission.*

2. Please provide separate figure files in .tiff or .eps format only, and remove any figures embedded in your manuscript file.  If you are using LaTeX, you do not need to remove embedded figures.

*Response: In the re-submission, the figure is uploaded separately in the formats stated.*

3. Tables should not be uploaded as individual files.  Please remove these files and include the tables in your manuscript file.

 Response: In the re-submission, the table is included in the manuscript.

4. In the online submission form, you indicated that "No additional or primary data will be made available due to the sensitive nature of the data and in alignment with the decision of the Ethical Review Board in Stockholm. This is to ensure the anonymity of the participants."

All PLOS journals now require all data underlying the findings described in their manuscript to be freely available to other researchers, either 1. In a public repository, 2. Within the manuscript itself, or 3. Uploaded as supplementary information. This policy applies to all data except where public deposition would breach compliance with the protocol approved by your research ethics board. If your data cannot be made publicly available for ethical or legal reasons (e.g., public availability would compromise patient privacy), please explain your reasons by return email and your exemption request will be escalated to the editor for approval. Your exemption request will be handled independently and will not hold up the peer review process, but will need to be resolved should your manuscript be accepted for publication. One of the Editorial team will then be in touch if there are any issues.

*Response:* Data cannot be made publicly available for ethical reasons. *Available data consists of 18 transcribed in-depth interviews. Even though names of persons/cities etc. are anonymised in the transcripts, the individual informants could be identified from the information provided in a complete interview transcript. In the application to the Ethical Review Board Stockholm, it was stated that the data should be handled with the highest respect for confidentiality. Further, we stated that the transcripts would only be shared among the co-authors. Thereby, we are not able to provide other researchers with our data. (This paragraph was submitted by e-mail on 2021-12-15)*

5. Please amend your detailed Financial Disclosure statement. This is published with the article, therefore should be completed in full sentences and contain the exact wording you wish to be published. i). State the initials, alongside each funding source, of each author to receive each grant.
ii). State what role the funders took in the study. If the funders had no role in your study, please state: “The funders had no role in study design, data collection and analysis, decision to publish, or preparation of the manuscript.”

*Response: Thanks. we have updated the information according to your guidelines and suggestion.*

Please review your reference list to ensure that it is complete and correct. If you have cited papers that have been retracted, please include the rationale for doing so in the manuscript text, or remove these references and replace them with relevant current references. Any changes to the reference list should be mentioned in the rebuttal letter that accompanies your revised manuscript. If you need to cite a retracted article, indicate the article’s retracted status in the References list and also include a citation and full reference for the retraction notice.

*Response: We have reviewed the reference list and we have found no errors. We have, aligned with helpful suggestions from Reviewer 1, added references to further strengthen the manuscript.*

REVIEWER 1

The is a well-written, thoughtful paper with important contributions towards helping improve our understanding of risk and pleasure among MSM. It could be strengthened by including intersectional analysis of how race, age, and other factors might affect perceptions of risk, and by making more concrete linkages to health promotion among MSM in the Discussion and Conclusion. The Introduction is strong, with an excellent exploration of the literature and outlining of key issues.

*Response: Thank you so much for insightful suggestions. We have addressed the given recommendations in the manuscript and your comments below.*

Can you try to bring in some global context at the beginning, to locate Sweden within the context of global health promotion on HIV/AIDS? E.g. why the choice of Sweden, how do efforts in Sweden compare to other settings, can you share data about prevalence in Sweden compared to other settings? Some statement on the state of existing qualitative research on MSM in Sweden might also be useful for the reader, to understand where the gaps lie.

*Response: Thank you for highlighting this issue. We have added text and references regarding the Swedish setting. The Swedish qualitative research on MSM and HIV is more than scarce and we have addressed this in the manuscript.*

Given the issue of pleasure arises in the results, it might be useful to foreground this a bit more in the introduction. Can you add additional literature on pleasure? Some suggestions may include Calabrese et al (2015) which focuses on race, Mabire et al (2019) which focuses on PrEP etc.

*Response: We fully agree with this comment and have addressed the issue in the manuscript.*

p. 7 – lines 150-152 – in the eligibility criteria, relationship status was not mentioned. Were all the men single? Were any married? This is quite important to clarify.

*Response: We agree, and this has been clarified in the manuscript.*

p. 7-8 – Berlin is mentioned a few times in the methods section. Can you explain the relevance of MSM’s presence in both settings? Is this a factor for a specific reason? The comparisons between Berlin and Sweden in the results would be clearer if this was explained earlier in the paper. Also, is there a reason it is always the city of Berlin compared with the whole country of Sweden?

*Response: The rational of choosing Berlin was more in-depth addressed in our first article (Dennermalm, et al 2019) and stemmed from HIV/STI data suggesting that Berlin was a city visited by a sub-group of sexually active Swedish MSM, but also a city of importance for MSM globally. For this manuscript, we aimed to step one step away from Berlin specifically and let the informants primarily represent a group of highly sexually active men. In other words, the whereabouts of the men, the Berlin context were, for the aim of this paper, of less importance.*

*Based on the HIV/STI data, we concluded that Berlin was the predominate German city for Swedish MSM to visit or live in. In contrast, if we had explored Spain, there would have been multiple cities included since Swedish MSM visit Barcelona, Madrid and Sitges among other places for exploring sexuality.*

In the methods section, some detail on ethics is needed apart from the ethics statement at the end of the paper. How was consent and confidentiality managed? Was data anonymised? Were referrals provided to participants who might have been distressed during the interview process, or who disclosed violence? How willing were participants to share details of other contacts and what ethical issues were associated with this sharing of information (referrals)?

*Response: We have elaborated on the procedure of ethics in the Method section to address the issues mentioned above. Our perception was that the informants appreciated the project and left the interview on a positive note despite the topic of HIV. The participants were helpful in recruiting new informants*.

Listing numbers in brackets after participant quotes is quite a clinical approach to presenting participant data which may be a bit dehumanising – can you find another way of presenting the information?

*Response: We understand and agree. We have changed the numbers to names to address this. The names are of course not the real names of the participants and this has been explained in the manuscript.*

Are there other factors that might influence MSM’s perspectives, that could be drawn out in the results. Race, economic status, migration status, education level and age are all factors that intersect to complicate and nuance MSM’s experiences and might result in different perceptions of risk. Can you take a more intersectional lens to draw out these aspects when presenting the data?

*Response: Intersectional perspectives have been discussed within the research group early on and throughout the project. There was a will to address race, economic status, migration status, education level, age, but also different facets’ of MSM, trans identities, disability/non-normative bodies. With less than 20 interviews in mind, an intersectional perspective providing room for all aspects mentioned above That said, we will address the lack of intersectionality in the Limitation section.*

p. 12 (line 259) – Did the participant describe this as being like the sword of Damocles or is this the authors interpretation? Can you clarify this statement?

*Response: Thank you for pointing this out to us. The term ‘sword of Damocles’ was not used by the informants but the wording was an interpretation by us. Re-evaluating the phrasing, we have decided to remove it.*

The conclusion could go a little further in articulating the implications for health promotion. Can you build on this more in the conclusion? The Discussion section would also benefit from greater discussion of the implications for public health messages globally. What implications might this have for work with MSM in settings like the US or UK?

*Response: Thank you for pointing this out. We have extended the discussion to address this.*

REVIEWER 2

This is a well developed manuscript.

Response: Thank you so much for acknowledging our efforts.

It would be useful to make use of the COREQ check list for qualitative data.

*Response: We agree and we have added the COREQ checklist with references to our text as an appendix to this letter.*

Either in the methods or the discussion, the authors should address the issue of reflexivity and how it was addressed either in data collection, analysis or interpretation.

*Response: Thank you for your suggestion. We have addressed this at the end of the Methods section.*
